# Synthetic Browsing Histories for 50 Countries Worldwide: Datasets for Research, Development, and Education

**DOI:** 10.1038/s41597-025-04407-z

**Published:** 2025-01-22

**Authors:** Dan Komosny, Saeed Ur Rehman, Muhammad Sohaib Ayub

**Affiliations:** 1 Department of Telecommunications, Brno University of Technology, Brno, Czech Republic; 2 College of Science and Engineering, Flinders University, Adelaide, Australia; 3 School of Science and Engineering, Lahore University of Management Sciences, Lahore, Pakistan

**Keywords:** Research data, Ethics

## Abstract

Browsing histories can be a valuable resource for cybersecurity, research, and testing. Individuals are often reluctant to share their browsing histories online, and the use of personal data requires obtaining signed informed consent. Research shows that anonymized histories can lead to re-identification, nullifying the anonymity promised by informed consent. In this work, we present 500 synthetic browsing histories valid for 50 countries worldwide. The synthetic histories are compiled based on real browsing data using a series of transformation criteria, including website content, popularity, locality, and language, ensuring their validity for the respective countries. Each history maintains the order of webpage accesses and covers a one-month period. The motivation for publishing this dataset arises from the community’s call for browsing histories from different countries for research, development, and education. The published synthetic browsing histories can be used for any purpose without legal restrictions.

## Background & Summary

There is a common need for large public collections of browsing histories for research, education, and development. However, making a large set of real browsing histories online poses high risks of privacy breaches and data misuse. Therefore, strict legal measures are in place to ensure that personal data are protected. Informed consent must be signed by each participant, which is problematic in large numbers and internationally due to different laws. Informed consent is common to ensure the anonymity of participants. This is an empty promise as individuals can be re-identified from anonymous browsing histories.

### Anonymity Questioned

Su *et al*.^1^ proved that anonymous browsing histories can be used to re-identify individuals. People commonly post URL links on their social media accounts. URLs posted on a person’s X account (previously known as Twitter) were used as the recommendation set *R*. The authors assumed that the user had visited the links in the recommendation set before their posting online. Therefore, these URLs are also present in the anonymous user history *H*. In the experiment carried out, 60 simulated users were identified by their profiles in 52 % of the cases using the maximum likelihood estimate. Other methods tested were the basic intersection size and the Jaccard index. The intersection method linked a person’s history with the recommendation set by most URLs present in | *R* ∩ *H* |. The Jaccard index was used to eliminate the effect of the size of the recommendation set | *H* ∩ *R* | / | *H* ∪ *R* |. The methods resulted in 42 and 13 % of identified users, respectively. The results showed that the maximum likelihood estimate outperformed the simpler methods. Deußer *et al*.^2^ showed that generalization to anonymize the data does not work. The authors attempted to re-identify persons from anonymous browsing data by a limited set of URLs known for the individuals. The known URLs may come from shoulder surfing, posted links on social networks, or general knowledge of the person’s browsing behavior. The authors used data from an audience measurement provider in Germany. Over 2.5 k websites and applications were included in the data. The anonymous dataset processed contained about 4.1 M clients, 1.2 k websites and 62 k endpages. The partially known data about the individuals were the webpages visited and time. The results showed that only 2 visited URLs with known access time in minutes are sufficient to link about 50 % of anonymous browsing sessions to individuals. 10 visited URLs are needed to link about 80 % of anonymous browsing sessions. With the URL visit timestamp less known, for example, as an hour or day, the identification was reduced substantially. However, 7 URL visits with the known day access time were needed to identify about 20 % of the anonymous browsing sessions.

### Informed Consent Needed

Informed consent is used to protect user privacy. It ensures that individuals who share their personal data are aware of the purpose and risks. Nature Editorials^3^ stated that research studies based on personal data need informed consent. Personal data is rarely used as originally intended. The outcome of the data processing can vary as unexpected patterns are often discovered. An example is given when phone tracking records were collected. The researchers could not have asked the participants to use their data for a purpose since the purpose was not known at the time of data collection. Therefore, informed consent must be relatively open to comply with unknown purposes that may cover research, education, and development. Furthermore, any participant should be able to withdraw their personal data at any time. This is almost impossible to implement in large amounts of anonymized data, especially when already posted online.

### Synthetic History Applications

The synthetic browsing histories in this dataset serve as a privacy-preserving alternative to real browsing data while offering significant potential for various research and testing applications. Synthetic browsing data can be used in cybersecurity research to train and test anomaly detection systems to identify suspicious browsing and overcome privacy restrictions of real-world data^4^. A specific example is phishing detection, as synthetic histories provide a valuable dataset of valid benign URLs for specific countries^5^. The benign URLs in the synthetic histories can be used to train phishing detection models. This would allow for tailored phishing detection systems. Another usage could be for the validation of machine learning algorithms, for example, to test web crawling, as the synthetic histories provide local URLs linked to places in countries^6^. Synthetic histories can also provide insights into global and local web data per country, including categories such as News, Shopping, and Business^1,2^. On the other hand, synthetic browsing histories cannot be used for behavioral insights by examining individual browsing data, including time-of-access patterns. The reason is that the owners of synthetic histories do not exist. The virtual owners of the histories mimic the behavior of the owner of the original history. The raw data were obtained through a web crawl from Common Crawl^7^. That means that the web content may be corrupted, retracted, or not accessible. Custom content filtering may be applied to synthetic history entries for specific applications.

### Limited Synthetic Web Data

There is limited work on synthetic web usage data. Hofgesang *et al*.^8^ proposed a methodology to build synthetic web data generators based on an extensive analysis of five real-world web usage datasets. Their approach emphasized the potential of synthetic data to bridge the gap in Web Usage Mining (WUM) research, where real-world datasets are often inaccessible due to privacy concerns. Their methodology relied on real-world usage patterns to create data generators that approximate actual web interactions. This enables the development of customer profiling and personalization techniques. However, it is limited only to five websites. Our work generates a large-scale dataset of synthetic browsing histories for 50 countries. The related efforts on synthetic data focused on domain-specific applications, including biomedical for assessing machine-learning healthcare software^9^, finance for predictive trading strategies^10^, fraud detection for the case of credit cards^11^, and generic education cases^12^. Unlike these efforts, our dataset is designed to support a wide range of research fields, including web analytics, recommendation systems, cybersecurity, and ethical AI development. We are using state-of-the-art tools such as Common Crawl to ensure the dataset includes real web data while safeguarding privacy.

## Methods

The synthetic browsing histories for 50 countries are based on a real browsing history. A series of transformation criteria are used to compile the synthetic histories for the respective country. The synthetic histories maintain the original history structure. The website access times in the synthetic histories are randomly shifted from the original history to ensure the privacy of the real history provider. The websites in the synthetic histories remain unchanged when used commonly in both the source and target countries. The website endpages in the synthetic histories are always transformed using the transformation criteria to maintain privacy. The transformation criteria used make each synthetic history valid for the target country. The compiled synthetic histories are verified for their validity in the target country.

### Base Algorithm

Algorithm 1 describes the compilation process of the synthetic browsing histories for the target country. The algorithm inputs are:

- Original browsing history. This is a real browsing history that is used to compile the synthetic browsing histories for countries.
- Common Crawl data. This data is used to transform the original history website endpages into the synthetic history website endpages. The websites and their endpages are transformed to be valid for the country.
- Country and world ranked websites. The most visited websites in the world and in the target country are used to transform websites from the original history to the synthetic histories.
- OpenStreetMap data. The mapping data are used to collect the local websites in the target country for the purpose of transformation of local websites, that is, websites that are not world or country ranked.
- Website content detection model. This model is used to estimate the content category of the websites in the original history and in the country transformation pools. The content category is used to match the same content-category websites between the original and synthetic histories.
- Language detection model. The language detection model is used to estimate the language of the websites in the original history and in the country transformation pools. The language is used to match the equivalent websites between the original history and the synthetic histories.
- Target countries. This is the list of countries for which the synthetic browsing histories are compiled.

#### Algorithm 1

Algorithm for compilation of synthetic browsing histories for countries.

Algorithm 1 lines 3-5 are explained in detail in the section ‘Target country data pools’. Lines 8-13 and 18-23 are explained in the section ‘Data classification’. Lines 28-43 and 48-54 are explained in detail in the section ‘Synthetic history transformation’.

### Target Country Data Pools

The country data pools contain websites and their endpages visited by users in the target country. These websites come from the country ranked websites, which are the top visited websites in the country. Country local websites are also included in the data pools. Local websites are visited by users in the country. Table 1 lists the created data pools for 50 target countries. The particular data sources used are the following:

- The country global websites are taken from DataForSEO^13^. DataForSEO provides lists of the top websites visited by users in the target countries.
- The country local websites are extracted from the mapping data provided by OpenStreetMap^14^. Websites related to places in the target country are used.
- The website endpages are compiled from Common Crawl^7^. Common Crawl provides extensive datasets of monthly collections from the whole Internet. The website endpages provided by Common Crawl from the same time range as the original history were used to ensure their time validity in the created country data pools.

**Table 1:** Country data pools for compilation of synthetic histories.

Country	Websites	Endpages	Country	Websites	Endpages
Argentina	126	2412	Austria	174	3433
Australia	113	2053	Belgium	162	3068
Bulgaria	133	2562	Brazil	91	1773
Canada	160	3007	Switzerland	177	3168
Chile	157	3134	Czechia	193	3858
Germany	194	3780	Denmark	170	3366
Estonia	165	3199	Egypt	118	2070
Spain	148	2815	Finland	219	4336
France	185	3708	United Kingdom	160	2996
Greece	98	1853	Croatia	216	4289
Hungary	214	4243	Ireland	172	3396
Israel	109	2024	India	165	3218
Italy	161	3141	Japan	143	2797
Sri Lanka	131	2445	Lithuania	184	3565
Latvia	145	2495	Morocco	89	1677
Mexico	141	2656	Malaysia	110	2106
Netherlands	198	3892	Norway	65	1215
New Zealand	135	2612	Pakistan	155	3061
Poland	204	4068	Portugal	136	2589
Romania	168	3270	Serbia	166	3270
Saudi Arabia	120	2083	Sweden	181	3614
Singapore	143	2694	Slovenia	176	3527
Slovakia	217	4463	Thailand	79	1456
Turkey	124	2453	Ukraine	73	1409
United States	158	3020	Vietnam	156	3069

### Data Classification

The classification is used for the website transformation from the original history into the synthetic history for the target country. The websites in the created country data pools and in the original history are classified for this reason. The classification process is the following:

- The websites are classified into top world ranks of 10, 100, 1000, 10000, 100000, and 1000000. The top 10 rank class means that the website is present in the 10 most world visited websites. The 1000000 rank means that the website is present in the 1000000 most world visited websites. A website that is not ranked is marked as −1. Majestic Million^15^ is used as the source data for the world rank classification.
- The websites are classified into top country ranks of 10, 100, and 500. The top 10 rank class means that the website is present in the 10 most visited websites in the country. The top 500 rank class means that the website is present in the 500 most visited websites in the country. A website that is not ranked is marked as –1. DataForSEO^13^ is used as the source data for the country rank classification.
- The website content is classified into the following categories: Business, Generic, News, Shopping, and Society. A machine-learning model by Lugeon *et al*.^16^ is used for content classification of the websites. Minor classes are joined in major groups. For example, the classes Sports and Games are covered by the major class Society. This ensures representative records for each group.
- The website language is estimated using a detection library provided by Stahl, P.^17^. The languages used for the target countries are the following: Hindi, Slovene, Turkish, Japanese, Bulgarian, Malay, Czech, Danish, Arabic, Nynorsk, Tamil, Polish, Thai, English, Irish, Serbian, Ukrainian, Hungarian, Slovak, Urdu, Vietnamese, Estonian, Swedish, Greek, Romanian, Croatian, Dutch, Latvian, Italian, Spanish, French, Finnish, Lithuanian, German, Portuguese, and Hebrew.
- The website endpages are classified into classes of 1, 25, 50 by the endpage path length. The first class is used for the root endpages of the websites. An endpage path longer than 50 is marked as –1.

### Synthetic History Transformation

A series of transformation criteria are used to compile the synthetic browsing histories for the target countries. The websites are first transformed, followed by their endpages. The access times in the synthetic histories are randomly shifted to mask the original history timestamps. The order of the webpage accesses is maintained as the original. Search websites in the original history are not processed, e.g., google.com. The original history is exported from a Google user account using the Google Takeout^18^ service available at https://takeout.google.com/settings/takeout in JSON format. Original history records are limited to a period of one month. The transformation steps are applied to the original history entries as follows:

1. Maintain the original history website if it is listed in the top country ranked websites (most visited websites) for the target country.
2. Filter websites from the same content-category in the target country pool as the original history website.
3. Filter websites from the same country rank in the target country pool as the original history website.
4. Filter websites from the same world rank category pool as the original history website.
5. Filter websites by the target country official language(s) pool or English.
6. Shift the access time of the original history endpage by a random value. The websites and their endpages access order are maintained as original.
7. Maintain the original history website if it is the root page.
8. Filter website endpages from the same path bin in the target country pool as the original website endpage.
9. Sample a result if there are more outputs from the matching criteria.

### Example History Record Transformation

An example transformation for a synthetic history record is presented. The original history record is https://www.bohemiapc.cz/pocitace-notebooky-prislusenstvi/komponenty/pameti/pameti-ddr5-pc/ with the access time 2024-11-29 20:42:33.421201. The target country for the transformation of the history record is Germany. The example processing steps refer to the pseudo-code lines in Algorithm 1. We do not skip lines with code structure to provide better reference to the algorithm.

1. Pseudo code line 27: This is the first line of original history transformation. The original history is iterated and the website record bohemiapc.cz is processed.
2. Pseudo code line 28: The original website bohemiapc.cz is checked for its presence in the target country ranked websites.
3. Pseudo code line 29: The condition about the website presence in the target country ranked websites is not met. The action of maintaining the original website in the synthetic history is not processed.
4. Pseudo code line 30: The original website bohemiapc.cz content category is checked. The original website content category is ‘Shopping’. There are websites with the same content category in the target country pool of Germany.
5. Pseudo code line 31: Websites with the same content category of ‘Shopping’ are filtered from the target country pool.
6. Pseudo code line 32: The original website bohemiapc.cz country rank is checked. The original website does not belong to the top visited websites in the country. It is therefore not a country ranked website.
7. Pseudo code line 33: The condition about the website presence in the top country visited websites is not met. The action of filtering websites with the same country rank from the target country pool is not processed.
8. Pseudo code line 34: Code structure line.
9. Pseudo code line 35: The original website bohemiapc.cz world rank is checked. The original website does not belong to the top word visited websites. It is therefore not a world ranked website.
10. Pseudo code line 36: The condition about the website presence in the top world visited websites is not met. The action of filtering websites with the same world rank from the target country pool is not processed.
11. Pseudo code line 37: Code structure line.
12. Pseudo code line 38: The original website bohemiapc.cz is checked for language. The result is ‘CES’. This language does not match the target country language, which is ‘DEU’.
13. Pseudo code line 39: The condition about the website same language used in the target country is not met. The action of filtering the websites with the same language from the target country pool is not processed.
14. Pseudo code line 40: Code structure line.
15. Pseudo code line 41: The original website language is not considered and the language of the target country is used. The target country pool contains websites only with the target country languages.
16. Pseudo code line 42: Code structure line.
17. Pseudo code line 43: A website from the filtered results of the target country pool is sampled. The result is the website https://www.berndwolf.de.
18. Pseudo code line 44: Code structure line.
19. Pseudo code line 45: Code structure line.
20. Pseudo code line 46: Code structure line.
21. Pseudo code line 47: The original website endpages are iterated and the endpage pocitace-notebooky-prislusenstvi/komponenty/pameti/pameti-ddr5-pc is processed.
22. Pseudo code line 48: The access time 2024-11-29 20:42:33.421201 is shifted by a random value to 2024-11-29 20:44:31.221766 to preserve privacy of the original history record.
23. Pseudo code line 49: The endpage pocitace-notebooky-prislusenstvi/komponenty/pameti/pameti-ddr5-pc is not a root page of the website.
24. Pseudo code line 50: The condition about the root page is not met. The action of maintaining the endpage is not processed.
25. Pseudo code line 51: The original endpage pocitace-notebooky-prislusenstvi/komponenty/pameti/pameti-ddr5-pc is checked for the path bin. The result is –1. There are websites with the same path bin in the target country pool.
26. Pseudo code line 52: Endpages with the same path bin are filtered from the target country pool.
27. Pseudo code line 53: Code structure line.
28. Pseudo code line 54: An endpage from the filtered results in the target country pool is sampled. The result endpage is antoinette-ketten-mit-anhaenger-sterlingsilber-925-granat-rot.

The final transformation result for the original history record https://www.bohemiapc.cz/pocitace-notebooky-prislusenstvi/komponenty/pameti/pameti-ddr5-pc with the access time 2024-11-29 20:42:33.421201 is the synthetic history record https://www.berndwolf.de/antoinette-ketten-mit-anhaenger-sterlingsilber-925-granat-rot with access time 2024-11-29 20:44:31.221766 valid for Germany.

## Data Records

The synthetic browsing histories are deposited on Figshare^19^. Table 2 describes the records of the published synthetic browsing histories. The core records are the access time and the URL. The extended records are the metadata of the original history used to compile the synthetic history record. Table 3 gives an overview of the values of each record. Table 4 shows the number of synthetic history records in the target countries. There are 10 synthetic histories compiled for each country.

**Table 2:** Core and extended records of synthetic browsing histories for countries.

Core records	
synthetic_time	Synthetic website endpage access time
synthetic_url	Synthetic website endpage

Extended records	
original_scope	Original website scope
original_content	Original website content category
original_country_rank	Original website country rank
original_world_rank	Original website world rank
original_lang	Original website language
original_bin	Original website endpage bin

Core records form the synthetic browsing histories. Extended records are metadata of the original history.

**Table 3:** Possible values of extended synthetic history records.

Extended record	Values
original_scope	Global, Local
original_content	Business, Generic, News, Shopping, Society
original_country_rank	10, 100, 500, −1
original_world_rank	10, 100, 1000, 10000, 100000, 1000000, −1
original_lang	Hindi, Slovene, Turkish, Japanese, Bulgarian, Malay, Czech, Danish, Arabic, Nynorsk, Tamil, Polish, Thai, English, Irish, Serbian, Ukrainian, Hungarian, Slovak, Urdu, Vietnamese, Estonian, Swedish, Greek, Romanian, Croatian, Dutch, Latvian, Italian, Spanish, French, Finnish, Lithuanian, German, Portuguese, Hebrew
original_bin	1, 25, 50, −1

None values are marked as −1.

**Table 4:** Unique websites and total records of synthetic histories.

Country	Websites	Endpages	Country	Websites	Endpages
Argentina	71	6186	Australia	84	6262
Austria	100	6283	Belgium	84	6258
Brazil	50	6125	Bulgaria	77	6332
Canada	120	6384	Chile	78	6252
Croatia	83	6104	Czechia	92	6194
Denmark	103	6308	Egypt	66	5771
Estonia	72	6041	Finland	116	5913
France	101	6172	Germany	103	5735
Greece	51	6088	Hungary	100	6336
India	101	6322	Ireland	116	6395
Israel	71	6262	Italy	91	6179
Japan	70	6244	Latvia	71	5798
Lithuania	83	6335	Malaysia	77	6414
Mexico	86	5775	Morocco	55	6082
Netherlands	121	6222	New Zealand	103	6441
Norway	39	6090	Pakistan	98	6282
Poland	104	6046	Portugal	69	5885
Romania	83	5614	Saudi Arabia	69	6205
Serbia	85	6407	Singapore	101	6299
Slovakia	81	6369	Slovenia	106	6327
Spain	73	6020	Sri Lanka	79	6127
Sweden	102	6282	Switzerland	99	6249
Thailand	40	6237	Turkey	53	6220
Ukraine	42	6034	United Kingdom	132	6392
United States	116	6439	Vietnam	64	6055

Table 5 shows sample values of a synthetic history for Slovakia. Extended samples of synthetic histories per country are available on GitHub^20^. Extended samples are present for each of 500 synthetic histories.

**Table 5:** Sample synthetic history records for Slovakia with metadata.

Synthetic	Synthetic	Original	Original	Original	Original	Original	Original
Access time	URL	Scope	Content	C rank	W rank	Lang	Path bin
2024-11-01 08:49:49.085098	https://www.gamesradar.com/age-of-empires-age-of-kings-review/	Global	Business	−1	10000	eng	50
2024-11-01 09:35:46.817464	https://www.digitaltrends.com/mobile/qualcomm-snapdragon-8-gen-1-plus-7-gen-1-xr/	Global	Society	−1	10000	eng	−1
2024-11-01 09:36:17.514660	https://www.birdz.sk/forum/mala-by-sa-europa-aktivne-zapojit-do-vojny-na-ukrajine/196669-tema.html	Global	Society	500	1000	eng	−1
2024-11-29 20:06:16.230158	https://www.tescoma.sk/zapekacia-doska-na-vafle-pre-sendvicovac-president-3-v-1-1-ks	Local	Shopping	−1	−1	ces	−1
2024-11-27 09:50:00.904429	https://aukro.sk/dalekohlady	Global	Shopping	100	100000	ces	25

## Technical Validation

The synthetic histories are technically validated using a series of verification tests. They are verified to contain websites commonly visited by users in the target countries. An empirical complementary cumulative distribution of the synthetic history websites among the country top visited websites illustrates their expected distribution. The content categories Business, Generic, News, Shopping, and Society are checked to be present in the synthetic histories for each country. An empirical complementary cumulative distribution of the transformed original history records shows that almost all the original history records were successfully transformed. The consistency in synthetic histories between countries is also verified.

**Fig. 1:**
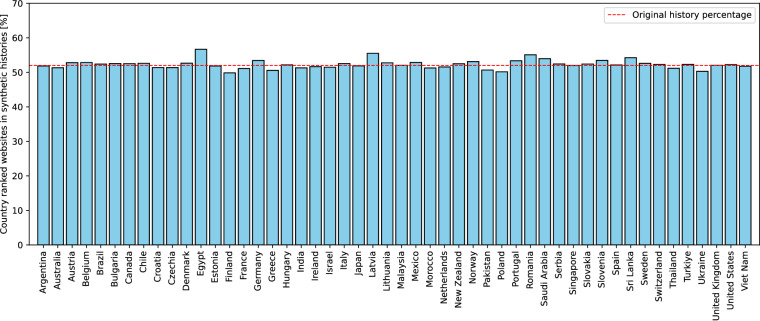
Percentage of synthetic history websites in countries that belong to the top country visited websites. Original history share is 52%.

**Fig. 2:**
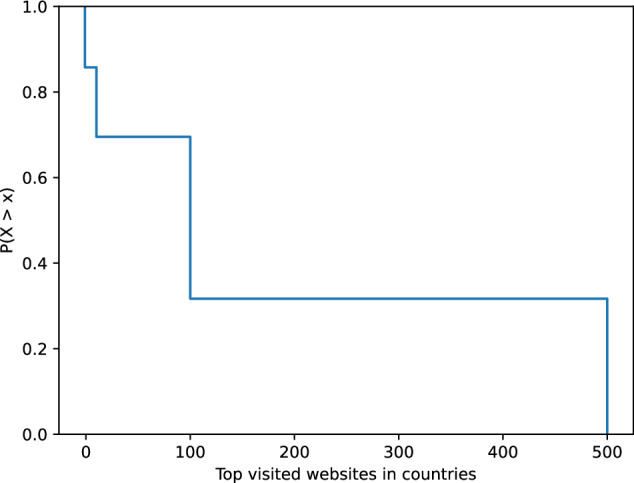
Empirical complementary cumulative distribution function of synthetic history records present in country top visited websites.

Figure 1 shows the percentage of synthetic history websites that are among the top websites visited by users in the respective country. The results show that the synthetic histories contain the top country websites in a proportion close to that of the original history, which is 52 %. The subtle differences between the synthetic histories are due to the transformation criteria. Figure 2 shows the empirical complementary cumulative distribution P(*X* > *x*) = 1 − P(*X* ≤ *x*) of the synthetic history records in the top visited websites in each country. The results show that the synthetic histories are present in the top 10, 100, and 500 visited websites in the country. The complementary cumulative distribution decreases with each top list, confirming the expected behavior.

Table 6 shows the distribution of the website content categories in the synthetic histories. The results show that all categories are present in the synthetic histories for the countries. Furthermore, the category records of Business, Generic, News, Shopping, and Society are consistent between the countries.

**Table 6:** Distribution of website content categories in synthetic histories.

Country	Business	Generic	News	Shopping	Society
Argentina	355	205	736	1264	3626
Australia	364	203	726	1277	3692
Austria	364	214	736	1377	3592
Belgium	360	217	729	1363	3589
Brazil	323	103	145	1349	4205
Bulgaria	313	199	2350	662	2808
Canada	345	205	733	1400	3701
Chile	347	206	732	1325	3642
Croatia	327	208	1562	1299	2708
Czechia	1698	212	149	1339	2796
Denmark	360	213	720	1376	3639
Egypt	343	212	1819	246	3151
Estonia	264	206	1499	1328	2744
Finland	324	186	1348	1284	2771
France	364	217	1727	323	3541
Germany	367	205	737	1090	3336
Greece	323	1510	149	1277	2829
Hungary	365	217	692	1412	3650
India	326	206	1585	1340	2865
Ireland	363	219	731	1369	3713
Israel	710	622	148	293	4489
Italy	368	187	728	1369	3527
Japan	484	372	270	296	4822
Latvia	319	206	1553	1347	2373
Lithuania	338	216	718	1399	3664
Malaysia	367	210	733	1377	3727
Mexico	362	199	736	1132	3346
Morocco	1017	171	1049	211	3634
Netherlands	365	213	711	1319	3614
New Zealand	637	743	731	334	3996
Norway	1384	214	1550	314	2628
Pakistan	358	213	738	1263	3710
Poland	327	196	1217	1502	2804
Portugal	364	195	1645	293	3388
Romania	330	210	149	2626	2299
Saudi Arabia	547	680	1123	323	3532
Serbia	360	205	707	1414	3721
Singapore	366	214	720	1354	3645
Slovakia	763	199	1152	1418	2837
Slovenia	365	208	731	1410	3613
Spain	758	498	467	191	4106
Sri Lanka	1405	123	727	320	3552
Sweden	363	211	689	1408	3611
Switzerland	367	197	735	1332	3618
Thailand	872	1667	674	198	2826
Turkey	318	1611	147	1399	2745
Ukraine	993	210	432	319	4080
United Kingdom	369	214	726	1389	3694
United States	368	216	734	1386	3735
Vietnam	329	193	1556	1271	2706

**Fig. 3:**
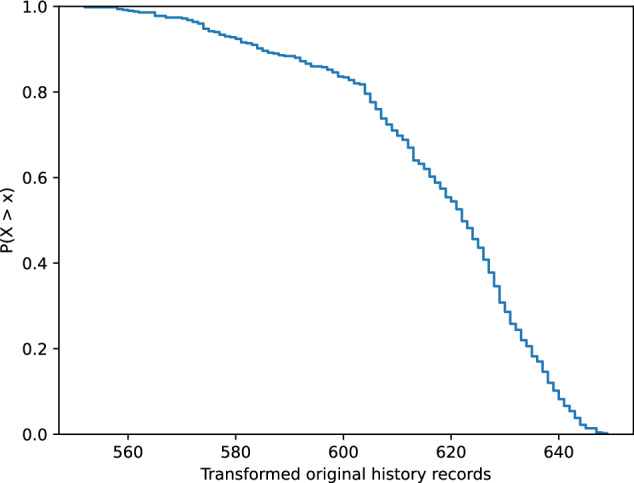
Empirical complementary cumulative distribution function of transformed original history records.

**Fig. 4:**
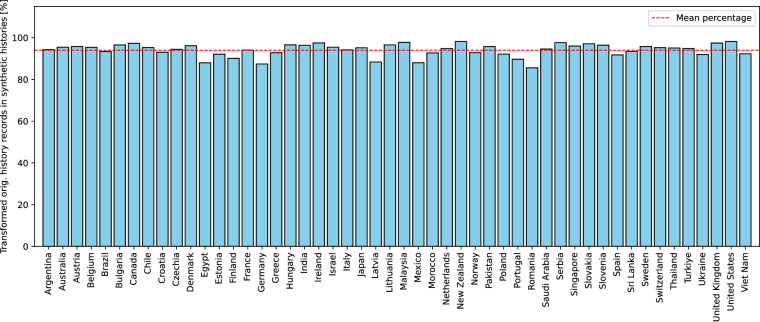
Successfully transformed synthetic history records in countries.

Figure 3 shows the empirical complementary cumulative distribution of the transformed original history records into the synthetic histories. The curve shows that the vast majority of the original history records was successfully transformed. The small number of records that could not be transformed was caused by their unsuccessful language detection. Figure 4 shows the consistency of the successfully transformed synthetic history records in the countries. The results show that the number of transformations is consistent across the countries with insignificant differences due to language detection.

## Data Availability

We publish a pseudo-algorithm that describes the transformation of the original history into the synthetic histories for the target countries. The form of the pseudo-algorithm ensures that personal data ethics are not violated. The pseudo-algorithm is described in detail to allow code implementation on original browsing histories. Furthermore, we also provide extended samples^20^ of synthetic browsing histories for better visibility and understanding^21^. Extended samples are provided for each of 500 synthetic histories. The authors offer to provide additional synthetic browsing histories based on their private original history. More synthetic browsing histories can be compiled in a reasonable number for the target country. The published synthetic browsing histories are available under the MIT License.
